# Use of sentiment analysis for capturing hospitalized cancer patients' experience from free-text comments in the Persian language

**DOI:** 10.1186/s12911-023-02358-2

**Published:** 2023-11-29

**Authors:** Azita Yazdani, Mohammad Shamloo, Mina Khaki, Azin Nahvijou

**Affiliations:** 1 Health Information Management Department, School of Health Management and Information Sciences, Shiraz University of Medical Sciences, Shiraz, Iran; 2 Health Human Resources Research Center, Shiraz University of Medical Sciences, Shiraz, Iran; 3 Clinical Education Research Center, Shiraz University of Medical Sciences, Shiraz, Iran; 4 Cancer Research Center, Cancer Institute of Iran, Tehran University of Medical Sciences, Tehran, Iran

**Keywords:** Sentiment analysis, Natural language processing, Topic modeling, Opinion mining, Cancer, Patient feedback

## Abstract

**Purpose:**

Today, the Internet provides access to many patients' experiences, which is crucial in assessing the quality of healthcare services. This paper introduces a model for detecting cancer patients' opinions about healthcare services in the Persian language, both positive and negative.

**Method:**

To achieve the objectives of this study, a combination of sentiment analysis (SA) and topic modeling approaches was employed. All pertinent comments made by cancer patients were collected from the patient feedback form of the Tehran University of Medical Science (TUMS) Cancer Institute (CI) in Iran, from March to October 2021. Conventional evaluation metrics such as accuracy, precision, recall, and F-measure were utilized to assess the performance of the proposed model.

**Result:**

The experimental findings revealed that the proposed SA model achieved accuracies of 89.3%, 92.6%, and 90.8% in detecting patients' sentiments towards general services, healthcare services, and life expectancy, respectively. Based on the topic modeling results, the topic "Metastasis" exhibited lower sentiment scores compared to other topics. Additionally, cancer patients expressed dissatisfaction with the current appointment booking service, while topics such as "Good experience," "Affable staff", and "Chemotherapy" garnered higher sentiment scores.

**Conclusion:**

The combined use of SA and topic modeling offers valuable insights into healthcare services. Policymakers can utilize the knowledge obtained from these topics and associated sentiments to enhance patient satisfaction with cancer institution services.

**Supplementary Information:**

The online version contains supplementary material available at 10.1186/s12911-023-02358-2.

## Introduction

The Web has become a rich data environment that provides a considerable amount of information with the cooperation of all Internet users without any restrictions [1, 2]. Today, patients and physicians are turning to online platforms such as blogs, social media, and healthcare websites to share their comments and leverage the perspectives of others on health issues [3]. Many unstructured data about healthcare quality are available on the Internet that have not been systematically recorded. Free-form text responses, when compared to structured questionnaires, may be less biased by the feedback collector and, therefore, more representative. However, analyzing large quantities and obtaining meaningful results becomes more challenging [4, 5]. Also, the enormous amount of news disseminated through social media makes manual verification unfeasible. Therefore, automatic analysis of online customer reviews, particularly the emotions expressed in comments, and fake news detection are considered hot research topics [6]. Natural language processing (NLP) is one of the technologies used in these fields. NLP is a branch of artificial intelligence (AI) that analyzes and understands human language. The goal of NLP is to design and build systems that can analyze human languages. One of the applications of NLP that data scientists highly regard is sentiment analysis (SA) [7]. SA is the field of study that analyzes people's opinions, sentiments, appraisals, evaluations, attitudes, and emotions towards entities such as products, services, organizations, individuals, issues, events, topics, and their characteristics. Sentiments can be positive, negative, or neutral and are assigned a numerical score indicating their effectiveness [8]. Today, many patients' experiences are available on the Internet [9]. SA allows us to understand and use this information more effectively to improve the quality of healthcare [10].

By leveraging SA, healthcare providers can gain valuable insights into their patients’ needs and preferences, allowing them to tailor their services and marketing strategies to better meet those needs. This can lead to increased patient referrals and higher revenue [5, 11]. Social media analysis provides the necessary capacity to monitor public health. Social media provides a platform for patients with chronic diseases, including cancer, to share their quality of life indicators with other patients during and after treatment [12].

Cancer is an important disease affecting millions of people and families worldwide [13]. For many reasons, such as the severity of some cases, the side effects of some treatments, and the deaths of other patients, cancer patients are often affected by serious emotional disorders such as depression [14]. Monitoring patients' mood is an important part of their treatment. Cancer patients use online social media and virtual cancer communities to exchange messages and support each other during treatment [15]. SA methods can be used to detect cancer patients' moods by analyzing their messages in online communities [16].

SA researches are expanding incredibly quickly. However, despite the fact that a sizable portion of the data is available in other languages, the majority of research efforts are focused on English language [17].

In the healthcare domain, most research efforts in the field of SA are focused on the English language [18, 19], while research is more limited to other languages including Persian.

SA in every language has specific prerequisites. Therefore, the direct use of methods, tools, and resources developed for the English language in Persian has limitations [20]. Therefore, it seems necessary to analyze patients' emotions in the Persian Language.

In this paper, we presented a model for understanding cancer patients' unstructured opinions about their care in the TUMS CI.

Even though there have been numerous studies on SA in the field of cancer [21–27], this is the first one to be carried out in Iran for this particular patient population in Persian language.

## Related works

This section discusses several kinds of research in the field of SA using either English or other languages in the healthcare domain.

Alexander et al. [18] performed SA and topic modeling on unstructured comments on the England patient feedback web service. The results of sentiment analysis showed that patient feedback can be accurately classified into positive or negative sentiments, and the topic modeling approach can be used to identify topics in patient reviews. Also, data from this website was used by Greaves et al. [10] to understand patients' unstructured opinions about their care. They used SA techniques to categorize online free text comments by patients as positive or negative descriptions of their health care. In this study, a model is presented to automatically predict whether a patient would recommend a hospital, whether the hospital is clean, and whether patients are treated with dignity. Their results show that, from free text, a logically accurate assessment of patients' views on various aspects of a hospital's performance can be predicted.

Clark et al. [12] conducted research on English-language tweets related to the experiences of breast cancer patients based on SA. The results of this study showed that patients shared positive experiences about treatment, increasing support and spreading awareness. The results of this study show that social media can provide a positive outlet for patients to talk about their needs and concerns regarding their healthcare coverage and treatment needs.

Balakrishnan et al. [21] examined the dynamics of emotions in patient narratives in a breast cancer community group to identify changes in emotions, thoughts, stress, and coping mechanisms during treatment options, particularly chemotherapy, radiation, and surgery. The sentiment dynamics of users' posts were analyzed using a deep learning model. A sentiment change analysis was performed to measure the change in users' satisfaction levels. This leads to a better understanding of the different needs of patients during treatment.

Edara et al. [19] provided a distributed framework for analyzing the sentiments of cancer patients from various online cancer support communities. In this study, a dataset was constructed by collecting patients' opinions from different domains using the Twitter API. The proposed distributed framework replaces traditional sentiment analysis approaches with a Long Short-Term Memory (LSTM) neural network for analyzing large volumes of data in a potential stream. The results of this study showed that most patients expressed positive opinions about their illness, while some expressed negative and neutral opinions.

In the study by Baker et al. [28], the response of individuals to colorectal cancer was analyzed and used to predict the future of this disease. The dataset for this study was collected from Twitter. LSTM models, Gated Recurrent Units (GRU), and Convolutional Neural Networks (CNN) were evaluated. The results showed that the GRU model outperformed the LSTM and CNN models in terms of accuracy and provided more stable results.

Modave et al. [22] used topic modeling and sentiment analysis techniques to understand discussion topics and quantify twitter users' perceptions and sentiments regarding breast cancer. Their results show that the attitude of ordinary people towards breast cancer changes from time to time.

Alamoodi et al. [29] examined the general sentiments and topics discussed during different waves of the COVID-19 pandemic in Malaysia. In this study, a lexicon-based approach was used to analyze sentiment in Twitter data. The results of topic modeling and SA showed a consistent presence of positive sentiments throughout all quarantine waves.

Pandesenda et al. [30] measured the service quality of mobile health services provided by Alodokter through the SA of customer comments in Indonesian from the Google Play Store. It is Indonesia's number-one digital health firm. System quality, interaction quality, and information quality are categorized using sentiment analysis and the Fast Large-Margin classification methodology. The findings of this study show that system quality receives the majority of good customer feedback, and interaction quality receives the majority of positive customer feedback.

Alayba et al. [31] used machine learning (ML) methods in the opinion classification to positive and negative classes. They introduce an Arabic language dataset in health services by collecting from Twitter.

Del-Arco et al. [32] used the Corpus Of Patient Opinions in Spanish (COPOS) and ML to mine Spanish patient opinions. They have carried out experiments with the main methodologies employed in the task of polarity classification (semantic orientation and machine learning).

Freedman et al. [33] used ML techniques to understand barriers to breast cancer treatment. They investigated the sentiments and barriers expressed about breast cancer treatments by Internet users.

The first study in the field of healthcare using SA and machine learning in the Persian language was done by Khaleghparast et al. [34] to identify various hospital wards and staff members from patients' remarks and to categorize messages into good and negative categories.

As a conclusion from previous related works SA in the field of health is presented on the comments and feedback published on different web platforms from health markets, and different social networks to the websites of healthcare centers with different goals and in different languages.

## Methodology

**Fig. 1:**
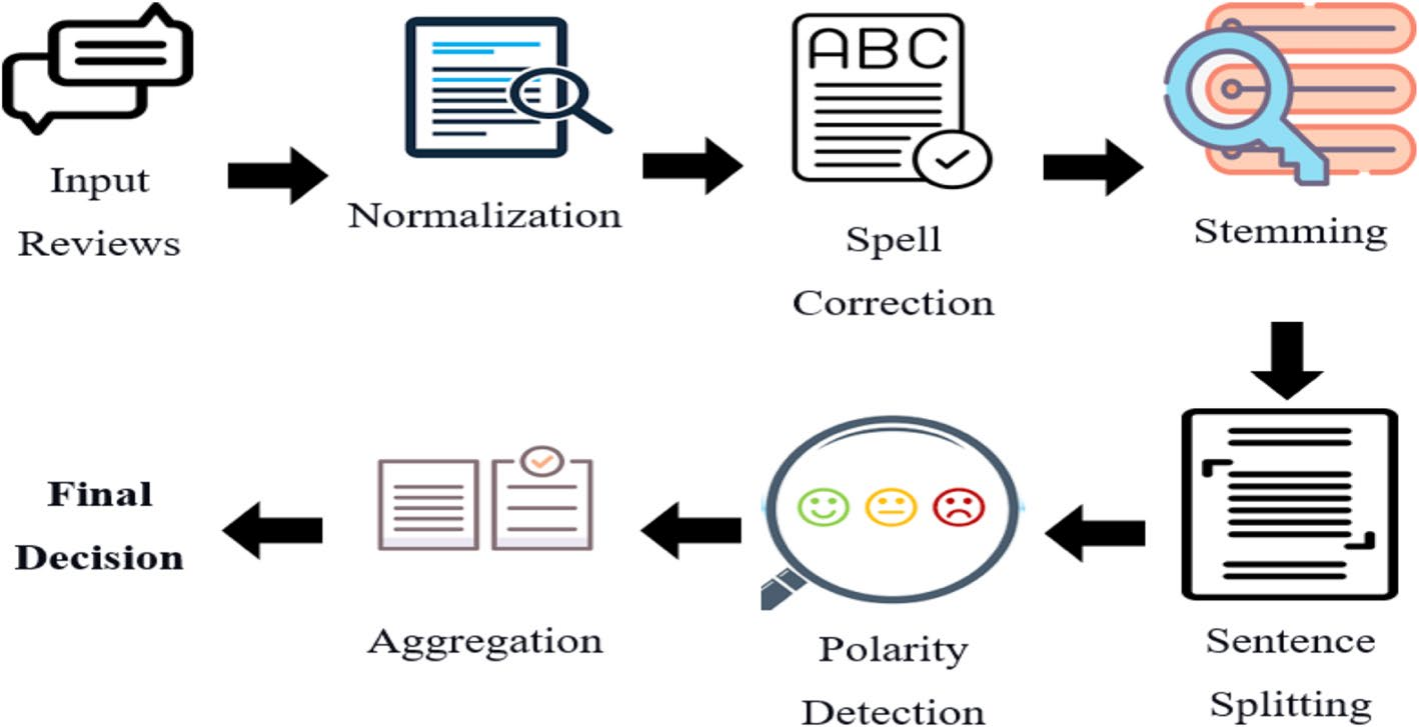
The block diagram of the research methodology

This work was part of a project evaluated and approved by the Ethics Committee under the number IR.TUMS.IKHC.REC.1399.377. All preprocessing and modeling steps were done in the Python version 3 environment. The block diagram of the research is shown in Fig. 1.

Each of the modeling steps is as follows:

- Normalization: If punctuation marks and Persian words are not written in the same way, the texts used will not be analyzable by computer systems. In the process of normalization, spelling signs, letters, spaces between words, abbreviations, etc. are converted to a standard form without making any meaningful changes to the text.
- Spell-checking: Spell checkers help quickly identify spelling errors in the text. This capability, if implemented properly, helps create texts without spelling mistakes and also increases the speed and accuracy of error detection.
- Stemming: In any language, words appear in different forms depending on their semantic and syntactic role in the sentence. This different form indicates the different meanings of these words, but since they are all derived from the same root, they will have relatively close meanings. For this reason, in many NLP and information retrieval applications, it is necessary to convert all derivatives of a word to its root.
- Sentence splitting: It involves the segmentation of a sentence into two or shorter sentences. It is a key component of sentence simplification, has been shown to help human comprehension, and is a useful preprocessing step for NLP tasks such as summarization and relation extraction.
- Polarity detection: It is employed to ascertain the sentiment of a text, indicating whether it is positive, negative, or neutral.
- Aggregation: Refers to a mechanism used to compute the overall sentiment score of reviews.

**Fig. 2:**
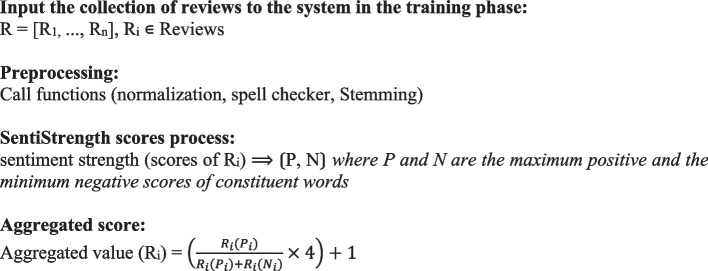
Pseudo code for the proposed model

Figure 2 illustrates a simplified representation of the proposed model using pseudocode, which is an informal method for describing algorithms without the need for a specific programming language [35].

### Data collection

All available cancer patients' feedback was collected between March and October 2021 from a patients' feedback form in the Tehran University of Medical Sciences Cancer Institute (TUMS CI), the most referred cancer hospital in Iran located in Imam Khomeini Hospital Complex. This institute is a leading organization in the field of cancer research and treatment. The TUMS CI consists of three divisions: education, research, diagnosis, and treatment. It was established in 1949 [36].

The patients' feedback form was prepared to receive the experiences of patients on the TUMS CI website.

Based on a literature review and the review of common complaints in Iranian hospitals, main categories were prepared to receive unstructured opinions from patients. The results of this step were reviewed in an expert panel session by five experts, including two medical informatics specialists, two healthcare services management specialists, and one oncologist, who had at least three years of experience at the Universities of Medical Sciences.

The nominal group technique (NGT) was used to evaluate search results. It allows stakeholders to directly produce items for needs assessment surveys [37]. Our goal was to use NGT discussions to develop online free text feedback items. Two NGT groups were conducted. Participants shared items, and a master list was compiled, and then reviewed by participants to remove or merge overlapping items. Once a final list was generated, participants independently rated them on a scale from 1 to 10. Lists generated in the NGT discussions were subsequently reviewed and integrated into a single list by research team members.

The final unstructured feedback form was made available online to cancer patients. They could provide free-text feedback about the TUMS CI in 3 main unstructured sections:

- Healthcare services in TUMS CI: Patients could provide feedback in this section about the treatment process, dignity, respect, staff cooperation, involvement in decisions, etc.
- TUMS CI's general services include an option for patients to describe their free-text experience with the accommodation environment, cleanliness, quality of food, and other relevant aspects in this section, etc.
- Cancer patients' life expectancy: Patients may express their free-text comments regarding their desire to continue their current treatment process and their concerns as cancer patients, etc.

These patients are free to feedback on each section. The patient feedback form helps us to collect valuable comments from patients about the clinic, hospital, their level of satisfaction, etc. All comments and reviews were stored in an Excel file for preprocessing and analysis.

### Preprocessing

The Persian language has particular challenges for which it is impossible to use many existing methods to analyze sentiments in English. One of the main challenges in Persian texts is the existence of words with different writing formats. Also, the existence of short space or pseudo-space as the space inside the word to separate words is one of the other challenges of the Persian language. Also, some letters in this language have different Unicode, such as "ی" and "ک" which has made marking and dividing texts in Persian more challenging than in other languages [38]. Before working on the texts to make the letters and spacing consistent, they must be pre-processed. At this point, all of the characters need to be replaced with their standardized counterparts. When dealing with Persian script, which is similar to Arabic script, there are often issues with the pronunciation of Arabic characters, including ("ک"، "ی") and ("ئ"، "أ" ،" ؤ"،" إ"). To rectify the issues related to these letters, the first step is to normalize them.

In Persian language, there is a module called hazm for normalizing the texts. It is a Python library for digesting Persian text [39]. In this research, the hazm library was used to carry out all preprocessing steps. A part of the codes can be seen in Additional file 1.

In the first stage, to prepare posts and comments, existing punctuation such as ("?"، "!"، "()") and so on were removed from the texts. Also, to reduce the comments' size and improve the accuracy, stop words that do not significantly affect the sentiment analysis process were removed. For this purpose, the list of existing Persian stop words previously suggested by Dolamic et al. [40] was used. In the next step, the stemming process was performed to reduce the different forms of words used in the comments [41].

The main problems of the Persian language that were solved in the pre-processing stage are as follows:

#### Different encoding for some characters:

Persian script processing often has issues when trying to use Arabic characters for certain characters. In the corpus, these characters have been standardized according to the Academy of Persian Language and Literature (APLL). APLL is the regulatory body for the Persian language, headquartered in Tehran, Iran [42].

#### Extra spaces

During the preprocessing stage, the text corpus was stripped of any extra spaces, half-spaces, and tabs.

#### Variety of text with uppercase and lowercase letters

In some cases, the letter "آ" is used incorrectly in words featuring it, such as writing "اب" instead of "آب". To resolve this issue, words beginning with the letter "ا" were altered to feature the uppercase form of the letter "آ". Additionally, words with the letter "آ" (other than their primary character) were changed to their lowercase form.

#### Different ways to add suffixes to main words

Various methods of appending suffixes to main words include the use of suffixes such as ("تر"، "ترین" ، "هایم" ، "هایش") and so on, which are placed at the end of the words in three different forms, including ("مناسبتر" ،"مناسب تر" ،"مناسبتر"). To make this type of words systematic, all suffixes are affixed to the end of the words.

#### Different ways of adding prefixes to main words

The prefixes of "می‌"، "نمی‌"، "درمی‌"، "برمی‌"، "بی‌" come in three different forms: "می رود" ،"میرود", and "می‌رود". To standardize this type of words, all prefixes should be added to the beginning of the words using a half-space.

#### Removing the character "-"

The character stretches out the words, such as "بــــــر" becoming "بر".

#### Different ways of attaching the components of compound words

A compound word is when two or more distinct words are combined to create a single, new term with a distinct meaning. For instance, the Persian word “پیرمرد” is composed of two separate words, “پیر” and “مرد”, each of which has its meaning. All the compound words from the corpus have been altered to one unified form.

#### Multiple spelling words

Persian words have multiple spelling forms. These words may contain homophones (ا/ ع ؛ ت/ ط ؛ ث/ س/ ص ؛ح/ ه ؛ ذ/ ز ؛ ض/ ظ ؛ غ/ ق) like "اتاق" and "اطاق", for which the correct form according to the APLL is"اتاق". Its reliable resources were used to verify and standardize multiple spelling words in the corpus.

#### The treatment of negations

Persian negation is very important because its structure is used in everyday conversation. Persian negation is the process that turns an affirmative statement (I am happy) into its opposite denial (I am not happy). In Persian, verbs are negated with the particle "نـ". This particle, being a word of its own, should be written separately but under the current custom of writing, it is written attached to the verb:

- رفتم (I went) → نرفتم (I did not go)
- خندیدم (I laughed) → نخدیدم (I did not laugh)

We use a rule-based approach to detect negation for sentiment analysis that largely draws information from lexicons. In this approach, every verb with the particle "ن ", "نا ", "ضد " (important shifters) is considered as negated [20].

### Sentiment analysis

In this study, to decide on the polarity of each review and comment, the method proposed by Basiri et al. [43] was used. It is based on the evidence theory of Dempster-Shafer and sentence-level sentiment aggregation. The polarity detection of each sentence was determined using SentiStrength and an existing library. SentiStrength is an algorithm that combines a lexicon-based approach with linguistic information and rules to handle negation, misspelling, punctuation, and emoticons. It was specifically developed to handle sentiment analysis in informal and concise English text [44]. We used the translation of SentiStrength for Persian which was presented in [43]. SentiStrength contained positive and negative terms based on the degree of influence from -5 to + 5. With the SentiStrength method, the maximum positive and minimum negative scores of a sentence are calculated to determine the overall polarity of an opinion. Based on this method, the maximum absolute value of the positive/negative score was considered the opinion score. This strategy calculated the opinion score based on the number of positive and negative sentences (Eq. 1).

#### Score aggregation strategies

In order to calculate the overall sentiment score of reviews, an aggregation mechanism is required:

1 $$\mathrm{V}(\mathrm{P})=\frac{\sum_{i=1}^{n}(u\left({T}_{i}\left(P\right)\right)\times Polarity\left({T}_{i}\left(P\right)\right))}{\sum_{i=1}^{n}u({T}_{i}\left(P\right))}$$

where V is the overall valuation (score) of a product P. It can be computed as a weighted average of the polarity of each individual review ${T}_{i}\left(P\right)$ where the weights indicate their relative usefulness [45]. This method computes the aggregated polarity of a product by ${u(T}_{i}\left(P\right))$. In other words, it computes the overall polarity as a simple average of individual review polarities.

### Topic modeling

Topic modeling is an unsupervised NLP technique where untagged documents are used to create a collection of topics represented by a list of words that occur most often in each topic. In this study, the latent Dirichlet allocation (LDA) topic modeling approach was used to categorize each comment into computer-generated topics. LDA first considered all topics obtained from documents as hypotheses and then tried to select topics that best represent the documents. LDA calculated the probability of a word for a given topic as well as the probability of a topic for a given document [46].

To choose the final value of the number of topics (k), several LDA models with different values of k including [5, 10], were created. Then the output of these LDA models was manually checked by evaluators. K should be set large enough to cover all possible topics with minimal overlap. Finally, k = 6 was selected.

### Model evaluation

The model's accuracy is evaluated by splitting the data into two parts, the training set and the test set. The training set is usually broken down even further, with one section used to train the model and another used to validate it. Parameters and models are tested and tuned using the training set until the highest predictive score is reached. The test set is then used to test the best model obtained. In this study, the most optimal result was attained with a composition ratio of 90:10.

The SA model performance was evaluated in terms of precision, sensitivity, specificity, and accuracy, F-measure (Table 1).

**Table 1:** The performance evaluation metrics

Measure	Formula
Precision	TP/(TP + FP)
Recall/Sensitivity	TP/(TP + FN)
Specificity	TN/(TN + FP)
Accuracy	(TP + TN)/(TP + TN + FP + FN)
F-measure	2*Precision*Recall/(Precision + Recall)

Table 2 shows the confusion matrix. The values of true positive (TP) and true negative (TN) give information when the classifier is doing data classification is true, while false positive (FP) and false negative (FN) give information when the wrong classifier is inside the performed data classification.

**Table 2:** Confusion matrix

		**Predicted class**	
		Positive	Negative
**Real class**	Positive	**True Positive (TP):** Patients’ message had positive label and the model classified it correctly	**False Positive (FP)**: Patients’ message had negative label and the model classified it to positive
	Negative	**False Negative (FN):** Patients’ message had positive label and the model classified it to negative	**True Negative (TN):** Patients’ message had negative label and the model classified it correctly

In addition, we use the area under the receiver operating characteristic curve (AUC) to evaluate the proposed model. It means how much the model is capable of distinguishing between classes.

## Result

Table 3 listed the number of comments and reviews and how many words and sentences were contained in each comment.

**Table 3:** The dataset content statistics

Number of comment	3600
Average number of words	41.3
Average number of sentences( in each comment)	3.9

**Fig. 3:**
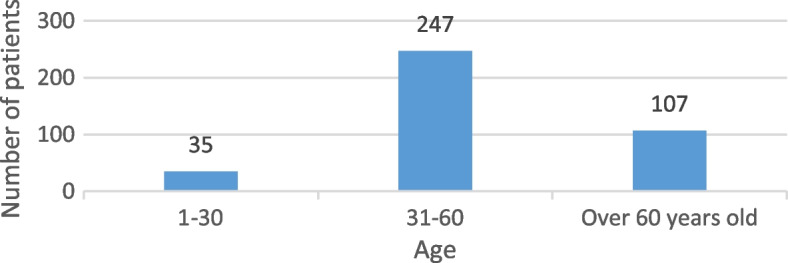
Age-frequency of patients participating in the free-text feedback form

The age frequency of the participants in the data collection phase is shown in Fig. 3. The highest frequency of participants was the age range of 30 to 60 years.

Table 4 shows a few examples from the corpus.

**Table 4:** Examples of comments

English translation	Comments in Persian
I am concerned about side effects after chemotherapy	.نگران عوارض بعد از شیمی درمانی هستم
The majority of the staff have appropriate and good behavior	.اکثر پرسنل درمان رفتار مناسب و خوبی دارند
The large number of accompanying patients causes excessive congestion in the treatment area	.تعداد زیاد همراه باعث شلوغی بیش از حد می شود

The patients' comments and reviews on each section were rated in one of the five predefined options by two experts, where 5 indicates the highest level of satisfaction and 1 indicated the lowest level of patient satisfaction. Two medical informatics experts rated the comments and reviews. In case of doubt, the sentiment was discussed and resolved between them.

For example, for the cleanliness of the hospital, there are five options: "completely clean", "very clean", "clean", "relatively clean", and "dirty". Also, the patients' comments and reviews related the dignity and respect were scored with the options of "always", "most of the time", "sometimes", "rarely", and "not at all". Regarding satisfaction with the treatment process, five options "completely hopeful", "hopeful", "relatively hopeful", "disappointed", and "completely disappointed" were used for rating. Comments rated 3, 4, and 5 were classified as positive, and comments rated 1 and 2 were categorized as negative.

**Fig. 4:**
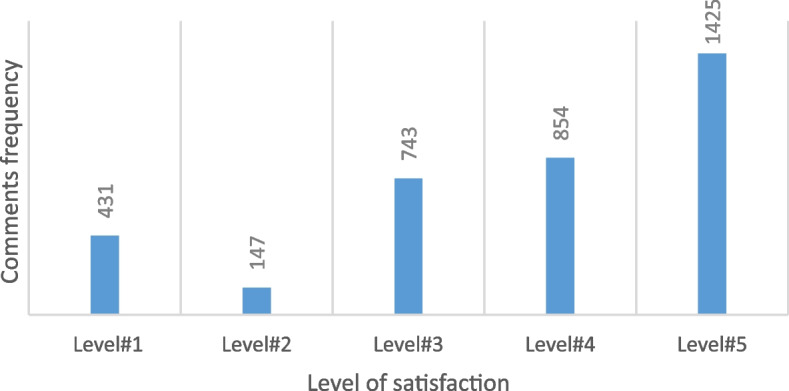
The frequency of categories of patients' comments based on the five levels of satisfaction

**Fig. 5:**
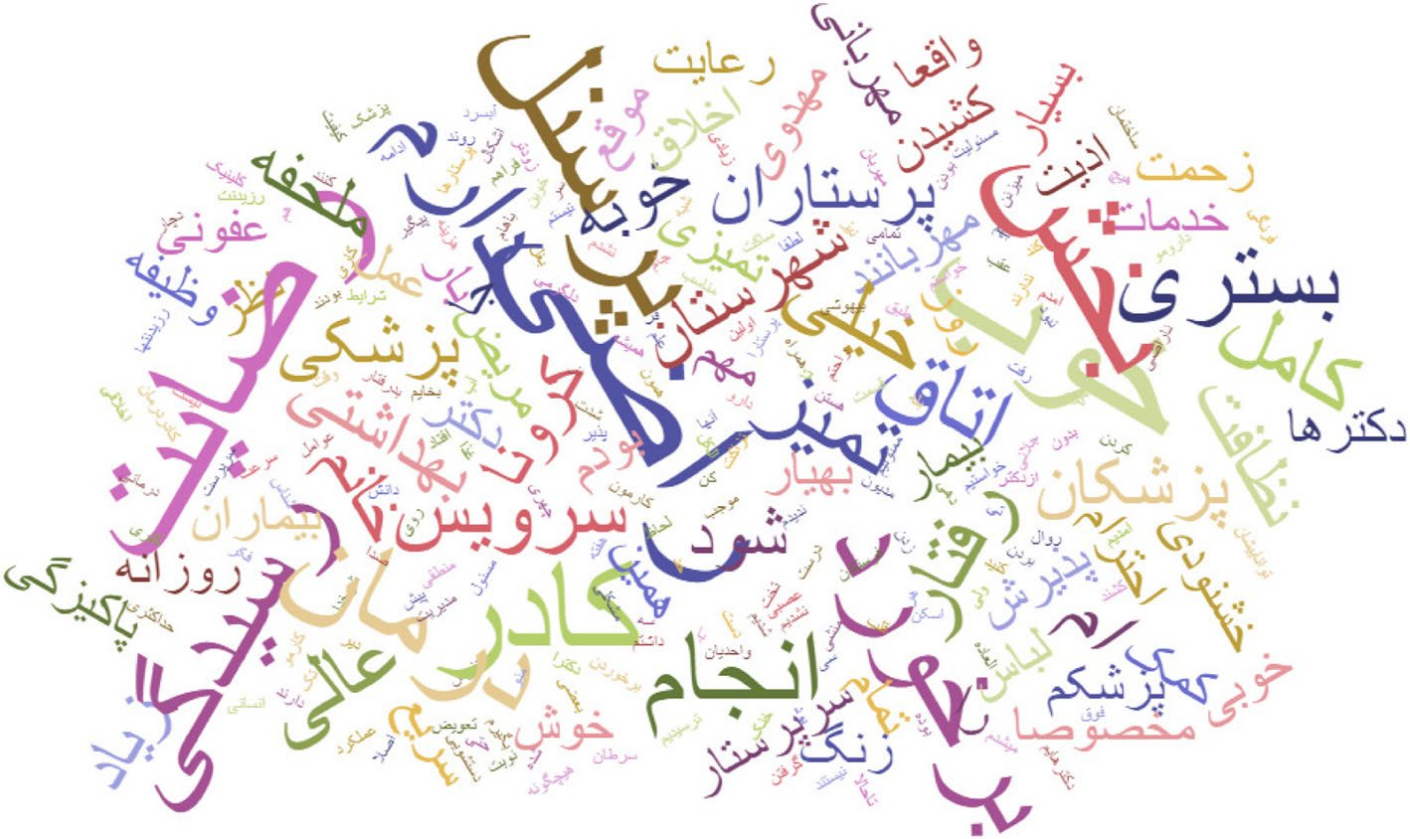
Persian words cloud of patients' comments about the healthcare services

According to Fig. 4, patients' satisfaction is evident in most of the comments. Also, by drawing words cloud on the comments related to the staff's behavior with the patients, the words "راضی ", "خوشحال ", "صمیمی ", "مهربان ", and "خوب " were the most repeated (Fig. 5). The English translation of each of the words respectively is equal to "satisfied", "glad", "friendly", "kind", and "good".

After applying the SA model to the comments and reviews, 89.3% of the quantitative ratings of patients' comments about "general services" and our prediction from the sentiment analysis were in agreement (Table 5).

**Table 5:** SA model performance

Metrics	General services	Healthcare services	Life expectancy
F-measure	93.1	95.1	94.1
Accuracy	89.3	92.6	90.8
Precision	91.4	94.3	93.2
Recall	94.9	93.5	92.4
Specificity	82.4	82.3	85.7
AUC	96.16	98.25	96.34

Similarly, SA on comments about "healthcare services" and "life expectancy" obtained 92.6 and 90.8% agreement between quantitative rating and sentiment analysis, respectively.

Table 7 reports the sentiment scores for each of the topics in each section. The topics identified by the LDA topic modeling approach were reviewed and labeled by AN, who is a GP in the CI. The analysis generated a score for positive or negative sentiment between − 1 (most pessimistic) and 1 (most optimistic). Examples of comments and their score can be found in Table 6.

**Table 6:** Frequency of topics and related sentiment score

Category	Top words	Classified reviews (number)	Sentiment score
healthcare services	Chemotherapy	10.2% (367)	0.253808
	Affable staff	5.3% (191)	0.331452
	Booking appointment	3.6% (130)	-0.161049
	Crowded room	1.4% (50)	-0.028327
	Cost and insurance	3.9% (142)	-0.108471
	Good experience	5.5% (198)	0.346670
general services	Tasty foods	3.1% (112)	0.175362
	Diet food	1.2% (43)	-0.001372
	Accompanying the patient	3.6% (130)	-0.125527
	Cleaned and Disinfected Environment	7.3% (263)	0.168454
	The bed sheet	4.1% (147)	-0.025697
	Air conditioning	2.2% (79)	-0.005819
	Glass	1.3% (46)	-0.002134
life expectancy	Metastasis	11.5% (414)	-0.304987
	Hopeful	18.2% (656)	0.125322
	Family	4% (145)	-0.025508
	Operations and surgery	2.4% (89)	-0.228316
	Complications of cancer	2% (74)	-0.024675
	Coronavirus	3.3% (121)	-0.117563
	Death	5.6% (203)	-0.121340
Total		100% (3600)	0.115483

**Fig. 6:**
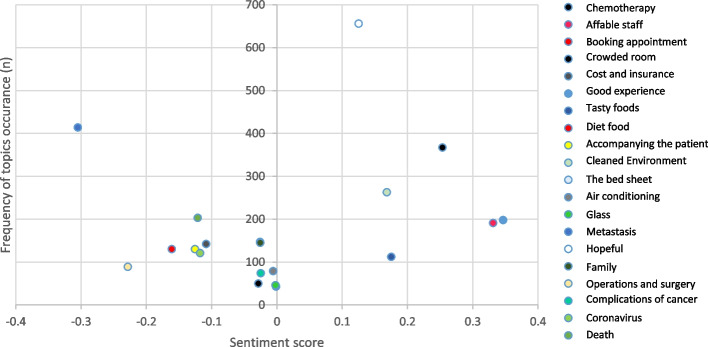
Relationship between topic frequency and sentiment

Topics that were innately associated with positive sentiments, such as "affable staff" and "good experience," have higher sentiment scores than inherently harmful topics such as a "crowded room" "good experience," "affable staff," and "chemotherapy" had higher sentiment scores compared with the other topics. "Metastasis" had lower sentiment scores than other topics (Fig. 6).

## Discussion

This study aimed to provide an automatic solution for analyzing cancer patient feedback about CI's healthcare services, general services, and life expectancy. This work examined NLP techniques, including a Lexicon-based method and topic modeling in the Persian language. SA caused automated analysis of patient comments. Also, the sentiment of comments was identified as positive or negative. The top 6 topics in all three categories were extracted through topic modeling. The experimental results showed that the patients' sentiments about healthcare services, general services, and life expectancy could be detected by the proposed model with an accuracy of 92.6%, 89.3%, and 90.8%, respectively.

In the category of life expectancy, "hopeful" as a positive word compared to "metastasis", "coronavirus", and "death" were the most frequent among patients' comments. The frequency of the words "cleaned & disinfected environment," "the bed sheet," and "accompanying the patient" was observed in comments related to general services. The sentiment score related to each topic showed that the patients are satisfied with the cleanliness of the cancer department, but they feel dissatisfied with their beds and the conditions of their companions.

Regarding healthcare services, "booking appointments" with the associated average sentiment score of -0.161049 mentioned that cancer patients might be unhappy with the current appointment booking service. Appointment scheduling was also an important determinant of patient satisfaction. Also, Alexander et al., in their study using SA and topic modeling on patients' experiences, introduced appointment booking as an issue that caused patients' dissatisfaction [18]. Studies showed that a patient's ability to book their non-urgent appointments significantly impacts patient satisfaction. Every day, people around the world strive to make their lives easier through technological advancements. No one anticipates wasting time, effort, and money waiting in line at the counter, especially when visiting a hospital. The development of an online appointment management system as a solution for patients’ online scheduling of hospital appointments with doctors based on their availability is helpful. These systems aimed to organize patient knowledge based on physician availability, hospital and specialist schedules, and patient appointments. These systems could be designed to automate daily hospital activities such as room activities, admission of the last patient, and doctor visits [47].

Cancer recurrence or metastasis was a predominant concern for many cancer patients and could contribute to experiencing a constant oscillation between feelings of hope and despair [48]. Metastasis was one the most frequent topics with the highest associated negative sentiment (-0.304987) in all three categories. It mentioned that recurrence or metastasis was a predominant concern for many cancer patients and could contribute to experiencing a constant oscillation between feelings of hope and despair. In a study by Mazza et al. [49] to investigate how to describe the lived experience of metastatic breast cancer on social media, they found that the effectiveness of treatment until recurrence or new metastasis was one of the concerns of cancer patients. Zhang et al. [50] focused on an extensive general breast cancer community and performed a sentiment analysis on all their online posts. They found that the posts published by older users were primarily about chemotherapy or metastasis/recurrence.

Accompanying the patient with the associated sentiment score of -0.125527 was the most negative sentiment in the general services category. It mentioned that cancer patients might be unhappy with the current services provided to their families and did not have a good experience. A good experience means that patients and their families would return to the hospital when they need more services and may even recommend the hospital to others. Hospitals today are designed with inspiration from hotels to increase the satisfaction of patients and their families. The concept of hospital hoteling is hospitality and non-medical services provided by patients and their families from the moment they enter the hospital until the final discharge, which could cause satisfaction or dissatisfaction. Hospital managers should be familiar with hoteling and have enough information to create strategies to improve health care services and patient satisfaction [51].

Social media usage is usually highest between 18–29-year-olds. Recent studies showed that the use of social networks in the age group over 65 has tripled in the past decade [49]. Examining the age group of participants in this study showed that 27.5% of the comments were related to people over 60 years old, which is comparable to people under 30 years of age, which was 8.9%. Therefore, patient feedback listening was a valuable technique for systematically reviewing the rich data that online platforms offer across the age spectrum.

Our results reinforce previous findings that it was possible to analyze the sentiments of patients' comments with a reasonable degree of accuracy and that it was possible to identify salient aspects of the comments [10, 52]. These results suggested a potential mechanism for using a large amount of text in the hospital complaint from where people describe their care and that further exploration of the information contained in free text comments may be an important way to understand the patient experience. It is considered an additional source of information to complete the traditional survey methods. The words expressing the patients' dissatisfaction and concern in the extracted free texts indicate the high noise level of the hospital environment, delay and crowding of appointments, high treatment costs, lack of support protocol for patients' companions, and problems in air conditioning systems. The extracted words express the patients' satisfaction with the free texts, show the proper behavior of treatment personnel, satisfaction with the cleanliness of the healthcare environment, and satisfaction with the food quality.

One of the main goals of any organization is to satisfy customers and clients by providing good quality services. Customer satisfaction is one of the leading indicators of growth and development. Client satisfaction is determined by speed, accuracy, precision in providing services, how clients are treated, and the availability of appropriate information. The developed model in this research provides the possibility of analyzing patients' opinions by recognizing the opinions of patients about different aspects. By receiving the opinions that were received through the online forms available in the complaints section of the hospital and also the comments provided on social networks, the proposed model could predict the sentiments of patients (positive and negative) according to the criteria of healthcare services, general services, and life expectancy.

As mentioned in Table 7, the existing SA methods include ML, deep learning, and lexicon-based methods [34]. In this research, a lexicon-based method was used.

**Table 7:** Comparison of the critical characteristics of prior works

Ref	Language	Coupes	Healthcare scope	Method	Objective /findings
# [18]	English	NHS website	Clinical service	NLP and Topic Modeling	- Classification of patients' emotions into positive and negative categories - Identifying frequent topics in patients' comments
# [10]	English	NHS website	Hospital's performance	ML	- Presenting a model to predict patients' views on different aspects of a hospital's performance
# [12]	English	Twitter	Breast cancer	NLP and ML	- Different experiences of the patient from the treatment process, their needs and concerns were identified
# [21]	English	Breast Cancer community group (Breastcancer.org)	Breast cancer	ML and Deep Learning	- The change in user satisfaction levels was measured - Changes in patients' emotions were investigated
# [22]	English	Twitter	Breast cancer	Deep Learning and topic modeling	- Identifying recurring topics - Identifying emotions over time
# [33]	English	Social networks, message boards, patient communities, and topical sites	Breast cancer	ML	- Understanding barriers to breast cancer treatment
# [30]	Indonesian	Google Play Store	Service quality	ML and Fast Large-Margin classification methodology	- Classification of service quality comments into positive and negative categories - Information quality, system quality, and interaction quality affect customer satisfaction
# [31]	Arabic	Twitter	Health services	ML and Deep Learning	- Classification of opinions into positive and negative categories
# [32]	Spanish	COPOS (Corpus Of Patient Opinions in Spanish)	Medical attention	Semantic orientation and ML	- polarity classification
# [34]	Persian	Database of Rajaie Cardiovascular Medical and Research Center	Hospital wards and staff members	Lexicon-based method and ML	- patients’ atisfaction analysis - Determining the different ward and staff names mentioned in patients’ messages
Our research	Persian	Patient feedback form of the Tehran University of Medical Science Cancer Institute	Hospitalized Cancer Patient	Lexicon-based method and Topic modeling	- patients' sentiments about general services, healthcare services, and life expectancy

SA in the field of health is presented on the comments and feedback published on different web platforms from health markets, and different social networks to the websites of healthcare centers with different goals and in different languages. In this research, we designed an unstructured patient feedback form, which will be the basis for receiving the online opinions of cancer patients on the TUMS CI website.

In the medical field, the purpose of SA and opinion mining is to augment healthcare services rendered to patients by evaluating their consent or discontent expressed in their comments. The amount of research that has been conducted in the field of SA of cancer patients is limited compared to other scopes.

There were few studies on SA in the Persian language [53–55] and so far, only one research [34] has been done in the field of healthcare. Our study is the first SA study conducted in the field of cancer in Iran. Compared to research [34] we also used a lexicon-based method but did not use ML methods. No topic modeling methods were used in their study. In both studies, the basis of the corpus was not the social network, but the information collected from the hospital's criticism and complaints form. In the corpus of the present study, similar to the study [34] patients' opinions about the care and non-care services of the hospital were analyzed. As a difference in our research, the life expectancy of cancer patients was also interpreted.

The corpus of this research is collected from the feedback recorded in the hospital's complaint and suggestions form, and the nature of the hospital's website is not a microblogging environment. So, to maintain the efficiency and scalability of the proposed model, the corpus should periodically feed from the recorded comments on the website.

## Limitations

Ironic sentences and words are one of the problems of SA, which is still considered an open challenge to develop tools for the English language. We also faced this problem in this research, which could be mentioned as one of the reasons for reducing the accuracy of the used model compared to the qualitative method. For example, the sentence "if the food were as good as the treatment staff, the situation would be wonderful" had a very high positive meaning from the system's point of view. According to this sentence, food quality is considered good from the system's point of view, but its meaning is negative.

The distinguishing feature of the research conducted in the corpus and the methods used. It is possible to compare the efficiency of the conducted research when they are all applied to the same corpus. Therefore, it was not possible to compare the accuracy of the current research with previous studies of the Persian language.

## Conclusion

By applying SA to healthcare, these analytical methods could interpret contextual information about the patient experience on a large scale. The results of this research could be used to identify patients' opinions about the current state of the cancer department. A combination of SA and topic modeling helps collect insightful information about healthcare services. Policymakers could use the learnings of these topics and related sentiments to improve patient satisfaction with cancer institution services.

In this research, only the unstructured experiences of the patients were analyzed. In future research, we would like to compare the results of SA with quantitative ratings provided by patients on a Likert scale to test the accuracy of prediction. For this, we will design a researcher-made structured questionnaire according to the goals of this research.

In the future study, we will develop a patient satisfaction analysis system based on the proposed model in this research. Considering that there was no unstructured form on the website of the TUMS CI and active social media that would meet the research objectives, we designed the complaints and criticisms form for the first time in line with the research objectives and distributed it to the patients. Considering the positive results of this research, in the future study, we will develop a patient satisfaction analysis system based on the proposed model in this research-based comments that will be gathered from the online designed form. There will be further studies and experiments on using deep neural network and recurrent neural network architectures to increase the accuracy of the results.

### Supplementary Information

**Additional file 1.** Some parts of the codes.

## Data Availability

The data generated, analyzed and used during the current study are not publicly available due to Shiraz University of Medical Sciences policy, but are available from the corresponding author upon the reasonable request.
