# Supplementary material for: Use of sentiment analysis for capturing hospitalized cancer patients' experience from free-text comments in the Persian language
Source: BMC Med Inform Decis Mak. 2023 Nov 29;23:275. doi: 10.1186/s12911-023-02358-2 (PMC10685532; doi:10.1186/s12911-023-02358-2)
Supplement: Supplementary file 1 — Additional file 1. Some parts of the codes. [file 12911_2023_2358_MOESM1_ESM.docx]

Additional file 1: Some parts of the codes

>>> from hazm import *

>>> normalizer = Normalizer()

>>> normalizer.normalize ( 'اصلاح نویسه ها و استفاده از نیم فاصله پردازش را آسان می کند' )

'اصلاح نویسه‌ها و استفاده از نیم‌فاصله پردازش را آسان می‌کند'

>>> sent_tokenize ( 'ما هم برای وصل کردن آمدیم! ولی برای پردازش، جدا بهتر نیست؟' )

[ 'ما هم برای وصل کردن آمدیم!' , 'ولی برای پردازش، جدا بهتر نیست؟' ]

>>> word_tokenize ( 'ولی برای پردازش، جدا بهتر نیست؟' )

[ 'ولی', 'برای', 'پردازش'، '،'، 'جدا', 'بهتر', 'نیست', '؟' ]

>>> lemmatizer = Lemmatizer()

>>> lemmatizer.lemmatize ( 'می‌روم' )

'رفت#رو'

>>> tagger = POSTagger ( model = 'resources/pos_tagger.model' )

>>> tagger.tag ( word_tokenize( 'سلام من را برسان.' ))

[

( 'سلام' , 'NOUN,EZ' ),

( 'من' , 'PRON' ),

( 'را' , 'ADP' ),

( 'برسان' , 'VERB' )

( '.' , 'PUNCT' )

 ]

>>> chunker = Chunker( model= 'resources/chunker.model' )

>>> tagged = tagger.tag ( word_tokenize( 'کتاب خواندن را دوست داریم' ))

>>> tree2brackets( chunker.parse (tagged))

'[کتاب خواندن NP] [را POSTP] [دوست داریم VP]'

>>> word_embedding = WordEmbedding (model_type = 'fasttext', model_path = 'resources/word2vec.bin' )

>>> word_embedding.doesnt_match ([ 'پنجره' , 'خداحافظ' , 'درود' , 'سلام' ])

'پنجره'

>>> parser = DependencyParser (tagger = tagger, lemmatizer = lemmatizer )

>>> parser.parse (  word_tokenize( 'زنگ‌ها برای که به صدا درمی‌آید؟' ))

<DependencyGraph with 8 nodes>
